# Phosphoenolpyruvate from Glycolysis and PEPCK Regulate Cancer Cell Fate by Altering Cytosolic Ca^2+^

**DOI:** 10.3390/cells9010018

**Published:** 2019-12-19

**Authors:** Juan Moreno-Felici, Petra Hyroššová, Marc Aragó, Sergio Rodríguez-Arévalo, Pablo M. García-Rovés, Carmen Escolano, Jose C. Perales

**Affiliations:** 1 Department of Physiological Sciences, School of Medicine, University of Barcelona, Feixa Llarga s/n, 08907 L’Hospitalet del Llobregat, Spain; juanmorenofelici@gmail.com (J.M.-F.); petra.hyrossova@gmail.com (P.H.); marc.arago@gmail.com (M.A.); pgarciaroves@ub.edu (P.M.G.-R.); 2 Laboratory of Medicinal Chemistry (Associated Unit to CSIC), Faculty of Pharmacy and Food Sciences, and Institute of Biomedicine (IBUB), University of Barcelona, 08028 Barcelona, Spain; sergio6_6@hotmail.com (S.R.-A.); cescolano@ub.edu (C.E.); 3 IDIBELL, Gran Via de l’Hospitalet 199, 08908 L’Hospitalet de Llobregat, Spain

**Keywords:** phosphoenolpyruvate carboxykinase (PEPCK), PEPCK-M, PCK2, phosphoenolpyruvate carboxykinase, cancer metabolism, calcium, c-Myc, NFAT, CaMK2G, phosphoenolpyruvate, TCA cycle, cataplerosis, sarcoplasmic reticulum Ca^2+^/ATPase pump (SERCA)

## Abstract

Changes in phosphoenolpyruvate (PEP) concentrations secondary to variations in glucose availability can regulate calcium signaling in T cells as this metabolite potently inhibits the sarcoplasmic reticulum Ca^2+^/ATPase pump (SERCA). This regulation is critical to assert immune activation in the tumor as T cells and cancer cells compete for available nutrients. We examined here whether cytosolic calcium and the activation of downstream effector pathways important for tumor biology are influenced by the presence of glucose and/or cataplerosis through the phosphoenolpyruvate carboxykinase (PEPCK) pathway, as both are hypothesized to feed the PEP pool. Our data demonstrate that cellular PEP parallels extracellular glucose in two human colon carcinoma cell lines, HCT-116 and SW480. PEP correlated with cytosolic calcium and NFAT activity, together with transcriptional up-regulation of canonical targets PTGS2 and IL6 that was fully prevented by CsA pre-treatment. Similarly, loading the metabolite directly into the cell increased cytosolic calcium and NFAT activity. PEP-stirred cytosolic calcium was also responsible for the calmodulin (CaM) dependent phosphorylation of c-Myc at Ser62, resulting in increased activity, probably through enhanced stabilization of the protein. Protein expression of several c-Myc targets also correlated with PEP levels. Finally, the participation of PEPCK in this axis was interrogated as it should directly contribute to PEP through cataplerosis from TCA cycle intermediates, especially in glucose starvation conditions. Inhibition of PEPCK activity showed the expected regulation of PEP and calcium levels and consequential downstream modulation of NFAT and c-Myc activities. Collectively, these results suggest that glucose and PEPCK can regulate NFAT and c-Myc activities through their influence on the PEP/Ca^2+^ axis, advancing a role for PEP as a second messenger communicating metabolism, calcium cell signaling, and tumor biology.

## 1. Introduction

Intracellular Ca^2+^ signaling is involved in the coordination of several cellular and physiological processes in normal and tumor cells alike, including bioenergetics, senescence [1], autophagy [2], apoptosis, cell proliferation, immune evasion, and metastasis [3,4]. The nuclear factor of activated T cells (NFAT) family of transcription factors is one of the key effectors of calcium signals in the control of immune system activation, inflammatory responses [5], angiogenesis, metastasis, and other biological processes [6]. Also, the proto-oncogenes of the Myc family of transcription factors involved in several cellular processes, including cell proliferation [7], and cell growth and metabolism [8,9,10], have been shown to respond to calcium both transcriptionally and post-transcriptionally upon CaMKIIγ-dependent phosphorylation at Ser62 [11].

Two principal calcium transporters and pumps regulate calcium trafficking between the ER and cytosol, IP_3_R, which consists on a Ca^2+^-permeable ER ion channel, and SERCA, a Ca^2+^/ATPase pump located in the ER membrane that regulate the return of Ca^2+^ from the cytosol to the ER lumen. SERCA activity is inhibited by oncogenes like Ras, and the antiapoptotic c member BcL-2, and activated by the tumor suppressor p53 [12]. Therefore, with DNA damage or cellular stress, calcium fluxes might determine cellular fate.

SERCA requires ATP to recover Ca^2+^ into the ER lumen, so under low energy conditions, SERCA activity can be compromised. In addition, glycolytic intermediates, such as glucose-6-phosphate (G6P) or phosphoenolpyruvate (PEP), can regulate SERCA activity in the brain [13] and T cells [14], independently of ATP. In T cells, extracellular glucose is key to activating cell proliferation, aerobic glycolysis, and anabolism as T cells mount anti-tumoral responses. Therefore, increased consumption of glucose by CD4 and CD8 T cells translates into raising the pool of PEP, inhibiting SERCA, and increasing cytosolic Ca^2+^ which in turn signals the activation of NFAT. In this context, an increment of PEP produced by overexpression of cytosolic PEPCK (PEPCK-C; PCK1) using retrovirus transduction was sufficient to inhibit SERCA activity and assure T cell activation [14]. However, the physiological role of the endogenous PEPCK pathway was not assessed. Interestingly, ER-stress up-regulates ATF4-dependent transcription of mitochondrial PEPCK (PEPCK-M; PCK2), an isoform of PEPCK commonly found in tumor cells [15]. In the tumor environment, high basal levels of ER stress are commonly found as stromal and cancer cells compete for limited quantities of nutrients [16]. In consequence, we hypothesize that the cataplerotic activity of PEPCK-M fluxing glutamine [17] and/or lactate [18] carbons towards the glycolytic intermediary pool might have a potential role in modulating cytosolic calcium signaling pathways, and hence the metabolic fate of the cell.

## 2. Materials and Methods

### 2.1. Cell Culture

Human colon (HCT116 and SW480) carcinoma cell lines were cultured in DMEM supplemented with 10% FBS, 10,000 units/mL penicillin, 10 mg/mL streptomycin, and 2 mM l-glutamine (Biological Industries, Kibbutz Beit-Haemek, Israel) and incubated in a humidified atmosphere of 5% CO_2_ at 37 °C. Ionomycin, PMA (phorbol 12-myristate 13-acetate), and Cyclosporin A were purchased from Merk (Darmstadt, Germany). 

### 2.2. RNA Extraction and Quantitative RT-PCR

Transcriptional regulation of targets of NFAT was analyzed by qRT-PCR at 6 h post-treatment. Total RNA was extracted using TRIsure^TM^ reagent. cDNA synthesis mRNA was performed using a high capacity cDNA reverse transcription kit (ThermoFisher Scientific, Waltham, MA USA). cDNA was quantitated using real-time quantitative RT-PCR assays in a 7900HT real-time quantitative RT-PCR system (ThermoFisher Scientific, Waltham, MA USA) using commercial Taqman primers for PTGS2 (Hs00153133-m1), IL6 (Hs00174131_m1), and TBP (Hs99999910_m1). Data analysis was based on the ΔΔCt method.

### 2.3. Western Blot

Cells were homogenized in RIPA buffer supplemented with protease and phosphatase inhibitors and centrifuged at 15,000× *g* for 15 min at 4 °C. Western blots were performed with 20–30 μg of cell extract. Proteins were separated in 8–12% SDS-PAGE and transferred to an Immobilon membrane (Merk Millipore, Burlington, MA, USA). Following primary antibodies were used: anti-phospho-Ser62/T58 c-Myc (Santa Cruz, Dallas, TX, USA; sc-377552), anti-c-Myc C-19 (Santa Cruz, Dallas, TX, USA; sc-788), anti-GLS1 (Abcam, Cambridge, UK; ab131554), anti-cSHMT A-2 (Santa Cruz, Dallas, TX, USA; sc-365203), anti-mSHMT F-11 (Santa Cruz, Dallas, TX, USA; sc-390641), anti-HK-2 (Cell Signaling, Danvers, MA, USA; 2867), anti-Glut1 (Santa Cruz, Dallas, TX, USA; sc-277228), anti-LDHA (Santa Cruz, Dallas, TX, USA; sc-137243). All membranes were normalized using mouse monoclonal anti-γ-tubulin antibody (Sigma-Aldrich, Darmstadt, Germany; T-6557). Horseradish peroxidase activity linked to secondary antibody was detected with ECL substrate (Pierce) in a Fujifilm LAS 3000 Intelligent Dark Box IV imaging system (Tokio, Japan).

### 2.4. Immunofluorescence

SW480 cells plated on coverslips (Ø 15 mm) were washed with PBS and fixed with 4% paraformaldehyde in PBS for 10 min. Cells were blocked in blocking buffer (PBS with 1% NHS, and 0.1% Triton^TM^ X-100) for 2 h and then treated with NFATc2 (A2) and c-Myc (C-19) primary antibodies (Santa Cruz, sc-514929 and sc-788 respectively) overnight at 4 °C. After 3 washes with blocking buffer, cells were incubated with anti-rabbit Alexa Fluor^®^ 555 (Invitrogen, Carlsbad, CA, USA, A28175) or anti-mouse Alexa Fluor^®^ 488 secondary antibodies (Invitrogen, A27039) for 2 h. During this incubation, DAPI was added to stain the nuclei. After washing 3 times with blocking buffer, samples were examined using a confocal laser scanning microscopy ZEISS LSM 880 (Carl Zeiss AG, Oberkochen, Germany) and ZEN 2012 (Zeiss, Oberkochen, Germany) was used to collect digital images.

### 2.5. Transfection and Luciferase Assays

Cells were transfected using polyethyleneimine (linear polyethyleneimine, Mr 25,000, Sigma-Aldrich, Darmstadt, Germany). The NFAT 3x-Luc plasmid (0.7 μg) and 0.3 μg of pSV40-β-galactosidase control vector (Promega, Madison, WI, USA) were co-transfected into 6-well plates containing 80% confluent cells and then distributed into 24-well plates. Cells were incubated overnight in complete medium before treatment. Luciferase activity was measured in a luminometer (TD 20/20; Turner Designs, San Jose, CA, USA) using the luciferase assay system (Promega). The luciferase values were normalized to β-galactosidase activity using the luminescent β-galactosidase detection kit II (Takara Bio USA, Kusatsu, Japan). pNFATx3-Luc vector was a gift of Mercè Pérez-Riba (Medical and Molecular Genetics Center, IDIBELL, L’Hospitalet del Llobregat, Spain). 

### 2.6. Cytosolic Calcium Measurement

Cells grown up to 80% of confluence in a 96-well plate were washed with PBS and then stained with Fluo-4 according to the manufacturer instructions (Molecular Probes^TM^ Invitrogen, Fluo-4 NW Calcium Assay Kit F36206). Fluorescence measurements were performed using the fluorescence spectrometer POLARstar Omega microplate reader (BMG LABTECH, Ortenberg, Germany).

### 2.7. Cellular PEP Loading

Cells grown up to 70% of confluence were washed and pre-incubated in sucrose medium (sucrose 250 mM, NaF 10 mM, glucose 10 mM, K_2_HPO_4_ 10 mM; pH 6.0) for 5 min. Then, cells were incubated for 15 min with sucrose medium with the desired PEP concentration. 

### 2.8. PEP Determination Assay

PEP was extracted with perchloric acid (1 M). PEP was determined through an enzymatic assay. The ATP formed during the conversion of PEP to pyruvate by pyruvate kinase was measured using StayBriteTM kit (Highly stable ATP bioluminescence assay kit K791-1000; Biovision, Milipitas, CA, USA). The samples were diluted in the PEP assay buffer (gly-gly 0.1 M; KCl 0.2 M; MgCl_2_ 1 mM; reconstituted enzyme from StayBrite^TM^ kit 0.1% (*v*/*v*); pH 7). The increment of luminescence was measured with a luminometer (TD 20/20; Turner Designs, San Jose, CA, USA) 2 min after the addition of pyruvate kinase (to a final concentration of 13.5 U/mL), and the results were normalized by protein content.

### 2.9. Glycolytic Flux Measurement 

Glycolytic flux was determined by measuring the formation of ^3^H_2_O from d-[5-^3^H]-glucose by the enolase step of glycolysis. Briefly, cells were treated with medium containing 1 μCi of d-[5-^3^H]-glucose/mL for 1 h at 37 °C. After incubation, triplicated 50 μL aliquots of media were transferred to uncapped PCR tubes containing 50 μL of 0.2 N HCl and placed into scintillation vial containing 0.5 mL non-labeled H_2_O. The sealed scintillation vials were left 24 h at 37 °C to allow equilibration between ^3^H_2_O produced by cells and non-labeled H_2_O. PCR tubes were removed and 10 mL of scintillation solution was added into each vial, mixed, and the radioactivity was quantified using a scintillation analyzer. The rate of glycolytic flux was corrected for recovery.

### 2.10. Glucose Consumption Assay 

Glucose consumption was determined by colorimetry assay, based upon two enzymatic reactions, catalyzed by glucose oxidase and peroxidase. The assay was performed by mixing 15 μL of media or standard with 200 μL of reaction buffer prepared as recommended by manufacturer PGO (Sigma-Aldrich, Darmstadt, Germany, P7119). Absorbance was measured at 450 nm after 30 min of incubation at 37 °C. The amount of consumed glucose was obtained by subtracting the final concentration values from the glucose concentration in the original medium. Finally, values were normalized by protein content. 

### 2.11. Lactate Production Assay 

Lactate production was determined by the measurement of NADH produced in the reaction catalyzed by lactate dehydrogenase enzyme (LDH). NADH was determined by fluorescence (excitation 340 nm/emission 460 nm). The assay was performed by mixing 10 μL of media or standard with 200 μL of reaction buffer (0.3 M hydrazine sulfate, 0.87 M glycine, 2.5 mM NAD^+^, and 0.19 M EDTA at pH 9.5). The background NADH fluorescence was measured and 25 μL of LDH (344 U/mL) was added into each sample. After 20 min of incubation at RT NADH synthesized in LDH, catalyzed reaction was measured and corrected for background. The amount of net lactate production was determined by subtracting lactate measured in the original medium. Finally, values were normalized by protein content.

## 3. Results

### 3.1. Glycolytic and Cataplerotic Fluxes towards PEP Cooperate to Regulate Cytosolic Calcium 

**Figure 1:**
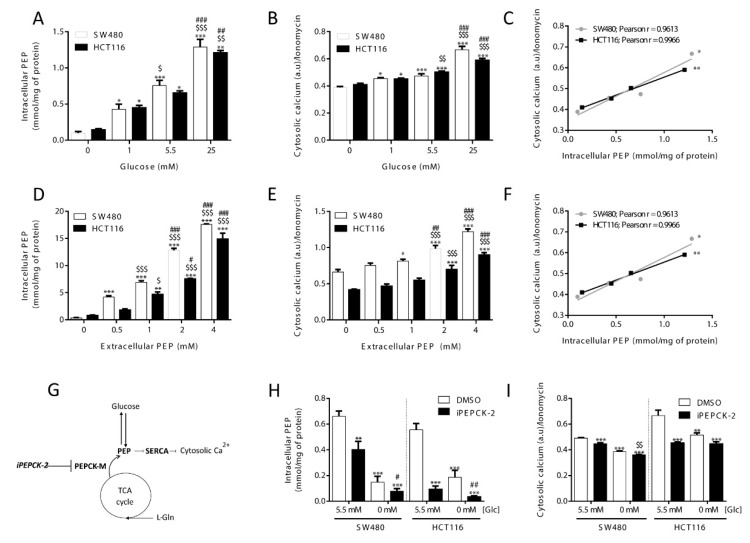
Glucose availability and PEPCK-M regulates cytosolic calcium through PEP. Intracellular levels of (**A**) PEP and (**B**) cytosolic calcium in both HCT116 and SW480 cells cultured on media with different glucose levels (**C**; correlation statistics). Effect of extracellular loading of PEP in both HCT116 and SW480 cells on (**D**) intracellular PEP and (**E**) cytosolic calcium concentrations (**F**; correlation statistics). Drawing depicting calcium regulation by PEP from glucose or cataplerotic sources (**G**). Intracellular levels of PEP (**H**) and cytosolic calcium (**I**) on both HCT116 and SW480 cells submitted to inhibition of the PEPCK pathway with 8.68 µM of IPEPCK-2 or vehicle (DMSO) at either 5.5 or 0 mM concentration of glucose in the media. Data are the mean ± SE, n = 5. One-way ANOVA, with Sidak post-test, *,^$^,^#^ *p* < 0.05, **,^$$^,^##^ *p* < 0.01, ***,^$$$^,^###^ *p* < 0.001. Correlation data was analyzed by a Pearson test. * versus 0 mM glucose or 0 mM extracellular PEP (A and B; D and E; D and E), or 5.5 mM Glc, DMSO condition (H and I). $ versus 5.5 mM glucose or 0.5 mM extracellular PEP (A and B; D and E), or glucose 0 mM, DMSO condition. ^#^ versus 1 mM glucose or 1 mM PEP (A and B; D and E).

The influence of glucose metabolism on the intracellular levels of PEP and its correlation with intracellular calcium was studied by measuring both PEP and relative calcium levels at different concentrations of extracellular glucose. As shown in Figure 1, cellular PEP linearly increased when glucose concentration in the media was elevated from 0 to 25 mM, both in HCT116 and SW480 colon carcinoma cells (Figure 1A). Cytosolic Ca^2+^ concentrations, as determined by relative Fluo4 fluorescence mediated by Ca^2+^ binding, correlated with the level of PEP and glucose media concentrations (Figure 1B; correlation statistics in Figure 1C). Loading known concentrations of PEP into cells caused a dose-dependent increase of Ca^2+^ (Figure 1D,E; correlation statistics in Figure 1F), providing further evidence that the glycolytic intermediate PEP can directly mediate changes in Ca^2+^ levels.

Cellular PEP levels are also maintained by the PEPCK-dependent cataplerosis of TCA cycle carbons that contribute to the synthesis of glycolytic intermediates (Hyrossova et al., unpublished), serine/glycine [19] and triglycerides [20,21]. In most tumor models, and specifically in the cellular models utilized in this work, the mitochondrial isoform of PEPCK (PEPCK-M) plays this key role as it is the only isoform of PEPCK expressed [22]. Therefore, we interrogated the implication of PEPCK-M in the regulation of PEP and Ca^2+^ fluctuations, we measured PEP and Ca^2+^ at various levels of activation of PEPCK-M more specifically using a potent inhibitor of PEPCK-M prepared in our laboratory, iPEPCK-2 [22], in the presence or absence of glucose (Figure 1G). PEPCK-M activity contributed to the PEP/Ca^2+^ axis in both colon carcinoma cell lines in the presence of glucose, as its inhibition effectively reduced cellular PEP concentrations and cytosolic calcium (Figure 1H,I). Glucose starvation was more effective at reducing PEP and Ca^2+^ levels than PEPCK-M inhibition, especially in SW480 cells. In the absence of glucose, the concentration of PEP was found close to the detection limit of our assay in both models but the inhibition of PEPCK-M was effective at further decreasing PEP concentrations (Figure 1H). All these results suggest a role for PEPCK-M in upkeeping the PEP pool, even in the presence of glucose. 

### 3.2. Intracellular PEP Modulates NFAT Activity and Its Target Genes

**Figure 2:**
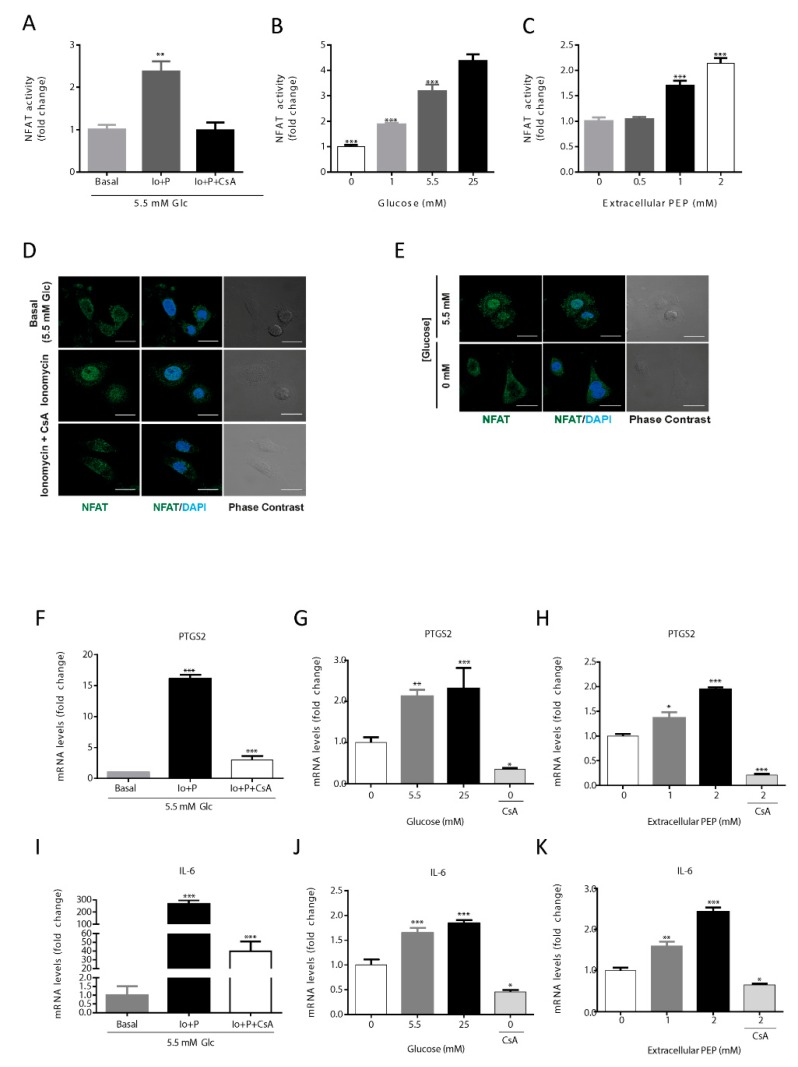
Activation of the NFAT pathway by PEP/calcium. NFAT is activated by ionomycin (1 µM) and inhibited by CsA (1 µM) (**A**). NFAT activity at various glucose concentrations in the culture media (**B**). NFAT activation levels at different extracellular PEP loads (**C**). Direct immunofluorescence for NFATc1 (green) and nuclear associated DAPI (blue) in basal, ionomycin (1 µM) and CsA (1 µM) conditions (**D**). Direct immunofluorescence for NFATc1 (green) and nuclear DAPI (blue) in the presence or absence of glucose in the culture media (**E**). NFAT target genes PTGS2 and IL-6 mRNA levels in the presence of ionomycin (1 µM) or ionomycin plus CsA (1 µM) (**F**,**I**). NFAT target genes PTGS2 and IL-6 mRNA levels at various glucose concentrations in the culture media (**G**,**J**). NFAT target genes PTGS2 and IL-6 mRNA levels at different extracellular PEP loads (**H**,**K**). Data are the mean ± SE, n = 5. One-way ANOVA, with Sidak post-test, * *p* < 0.05, ** *p* < 0.01, *** *p* < 0.001. * versus basal, 0 mM glucose or 0 mM extracellular PEP (A–C; F–K). Scale bar represents 20 μm.

To study if the presence of glucose, via modulation of PEP levels, can activate the calcineurin/NFAT cascade, we evaluated the activity of a luciferase reporter vector under the control of a chimeric promoter containing 3 adjacent canonical NFAT binding sites. The model was validated using ionomycin as a positive control of activation of NFAT, and cyclosporin A, to abrogate Ca^2+^ signaling (Figure 2A). With this tool, we analyzed the level of activation of NFAT at various glucose concentrations (Figure 2B) and upon PEP loading (Figure 2C). Increasing extracellular glucose or PEP concentrations produced a similar dose-dependent increase in NFAT activity. Furthermore, NFAT cellular localization was consistent with its measured activation level as it was preferentially located into the nucleus upon ionomycin treatment or in the presence of glucose and excluded into the cytosol when glucose was absent or calcium signaling inhibited with cyclosporin A (CsA; Figure 2D,E). 

Under NFAT activation, its target genes, IL6 and PTGS2, were transcriptionally up-regulated as analyzed by qRT-PCR (Figure 2F–K). Results showed that ionomycin-induced calcium flux significantly increased IL6 and PTGS2 gene transcription (Figure 2F,I). The expression of PTGS2 and IL6 were increased at higher concentrations of glucose (Figure 2G), being highest at 25 mM, and lowest at 0 mM glucose co-treated with CsA (Figure 2G,J). When PEP was loaded onto cells in culture, IL6 and PTGS2 mRNA content augmented in a dose-dependent manner (Figure 2H,K). Finally, CsA was able to abolish the up-regulation of IL6 and PTGS2 caused by PEP, demonstrating calcineurin/NFAT-dependent regulation (Figure 2H,K).

Collectively, these data indicate that NFAT dependent transactivation activity depends on glucose availability and subsequent intracellular changes on the PEP pool. 

### 3.3. Calcium-Dependent Phosphorylation at Ser62 Stabilizes c-Myc

**Figure 3:**
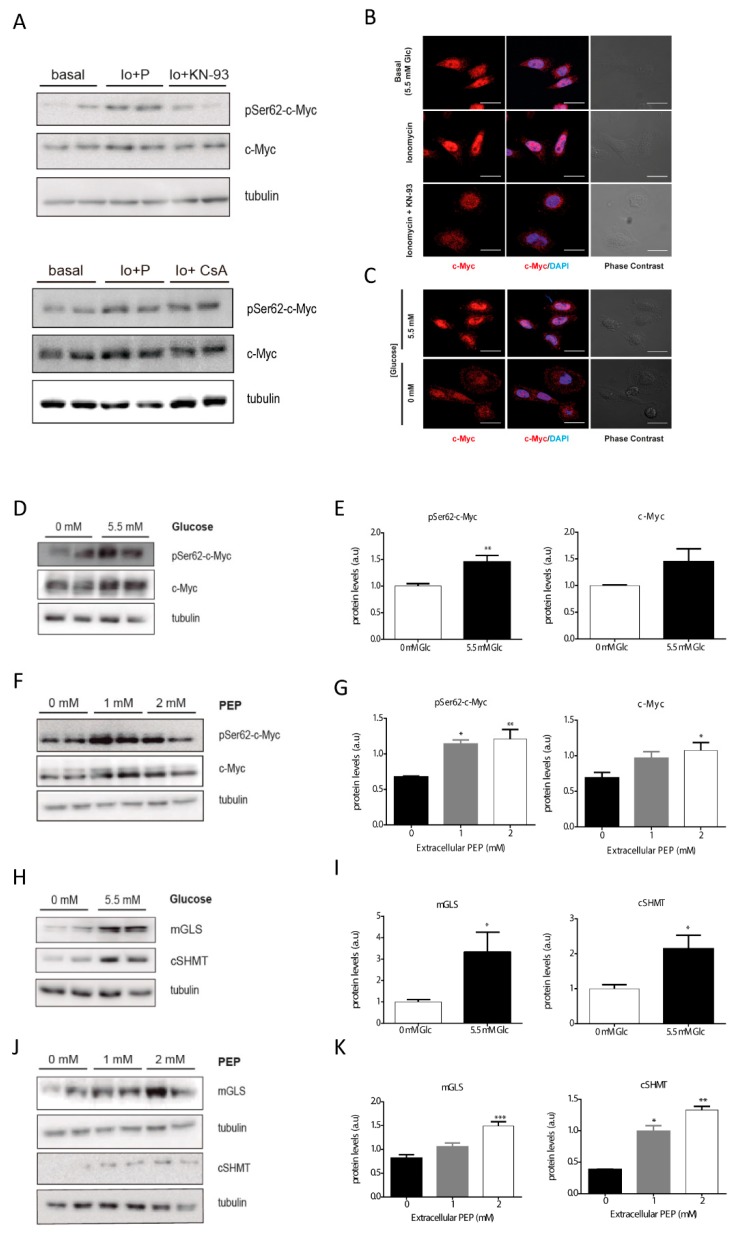
Glucose increases c-Myc phosphorylation at Ser62 in a PEP/Ca^2+^ dependent manner. Analysis of the phosphorylation of c-Myc by Western blot after 2 h in basal media (5.5 mM glucose), ionomycin (Io; 1 µM), ionomycin plus CsA (1 µM), and ionomycin plus KN-93 (5 µM) conditions (**A**). Direct immunofluorescence for c-Myc (red) and DAPI (blue) after 30 min treatment in basal, ionomycin (1 µM) and ionomycin plus KN-93 (5 µM) conditions (**B**). Direct immunofluorescence for c-Myc (red) and DAPI (blue) in the presence and absence of glucose in the media (**C**). Western blot analysis of the phosphorylation of c-Myc at Ser62 after 2 h in the presence of various concentrations of glucose in the culture media (**D**). Densitometric analysis (**E**) of WB shown in (D). Western blot analysis of the phosphorylation of c-Myc at Ser62 after 15 min treatment with extracellular PEP (**F**). Densitometric analysis (**G**) of WB shown in (F). Western blot analysis of mitochondrial glucosamine (mGLS), and cytosolic serine hydroxymethyltransferase (cSHMT), both described as a c-Myc targets, after 4 h in the presence or absence of glucose in the culture media (**H**). Densitometric analysis (**I**) of WB plotted in (H). Western blot analysis of mitochondrial glucosamine (mGLS), and cytosolic serine hydroxymethyltransferase (cSHMT), both described as c-Myc targets, after 15 min treatment with extracellular PEP (**J**). Densitometric analysis (**K**) of WB shown in (**J**). All experiments were performed in the SW480 cell line. Data are the mean ± SE, n = 4 (for western blot analysis, 2 replicates were loaded in each experiment, and each experiment was independently repeated at least twice). * *p* < 0.05, ** *p* < 0.01, *** *p* < 0.001; versus glucose 0mM or PEP 0 mM. One-way ANOVA, Sidak post-test. Scale bar represents 20 μm.

In T cell lymphoma (TCL) c-Myc protein is stabilized by calmodulin (CaM) kinase II gamma (CaMKIIγ) upon phosphorylation at Ser62 [11]. Therefore, we checked the phosphorylation status of c-Myc by Western blot in conditions that altered PEP and calcium pools. Ionomycin-dependent phosphorylation of c-Myc was shown with p-Ser62 specific antibodies (Figure 3A; quantitated in Supplementary Figure S3). Furthermore, phosphorylation was abrogated by pretreatment with KN-93, an inhibitor of calmodulin (Figure 3A; quantitated in Supplementary Figure S3). In addition, we assayed the subcellular localization of total c-Myc (Figure 3B). Total c-Myc increased its nuclear localization with ionomycin. These data suggest that proto-oncogene c-Myc is regulated by calcium signaling in cancer colon carcinoma cells.

As calcium signaling can stabilize the c-Myc protein, and glucose controls cytosolic Ca^2+^ levels through PEP, we next questioned whether the presence of extracellular glucose or PEP at varying concentrations might alter c-Myc phosphorylation and nuclear localization. Partial nuclear exclusion of c-Myc was observed under glucose deprivation (Figure 3C), in agreement with lowered phosphorylation observed under glucose starvation, a reduction that was mimicked by total c-Myc (Figure 3D; quantified in Figure 3E). Similarly, phosphorylated c-Myc was increased after two hours of treatment with PEP (Figure 3F; quantified in Figure 3G).

In addition, we evaluated the effect of glucose and PEP on the expression of c-Myc targets such as glucose transporter (GLUT1), mitochondrial glutaminase (mGLS), and the cytosolic serine hydroxymethyl-transferases (cSHMT; Figure 3H,J; quantitated in Figure 3I,K).

### 3.4. PEPCK-M Modulates Calcium Signaling Because of Its Impact on the PEP Pool

**Figure 4:**
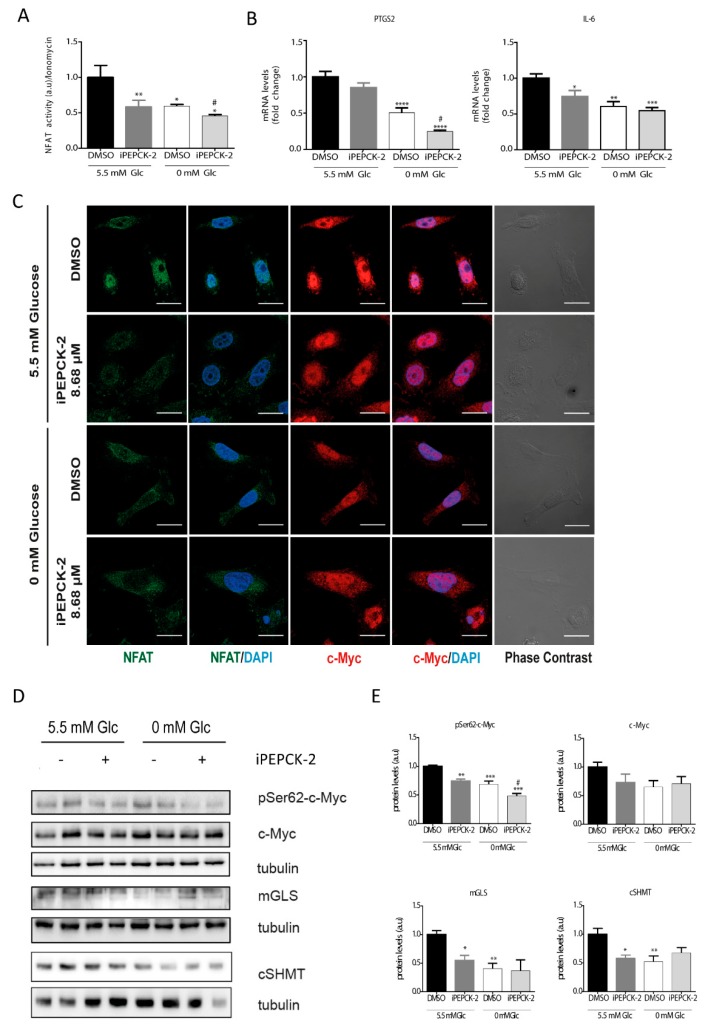
Consequences of PEPCK-M inhibition on the levels of activation of the NFAT and c-Myc pathways. (**A**) NFAT activity in the presence of IPEPCK-2 (8.68 µM) or vehicle (DMSO) in SW480 cells cultured in media with 5.5 mM glucose or the absence of glucose (0 mM). (**B**) PTGS2 and IL6 mRNA expression in the presence of IPEPCK-2 (8.68 µM) or vehicle (DMSO) in SW480 cells cultured in media with 5.5 mM glucose or the absence of glucose (0 mM). (**C**) Direct immunofluorescence for c-Myc (red) and NFAT (green) after 2 h of treatment with DMSO or IPEPCK-2 (8.68 µM), in SW480 cell culture in the presence or absence of glucose. (**D**) Western blot analysis of the phosphorylation status of c-Myc at Ser62, and the activation of several c-Myc target proteins. (**E**) Densitometry of WB shown in (**D**). Data are the mean ± SE, n = 4 (for Western blot analysis, 2 replicates were loaded in each experiment, and each experiment was independently repeated at least twice). * *p* < 0.05, ** *p* < 0.01, *** *p* < 0.001 versus DMSO/5.5 mM glucose; or ^#^ *p* < 0.05, ^##^ *p* < 0.01, ^###^ *p* < 0.001 versus DMSO/0 mM glucose. One-way ANOVA, Sidak post-test. Scale bar represents 20 μm.

As shown in Figure 1, PEPCK-M plays an important role in the maintenance of the PEP pool, even in the presence of glucose. In addition, we have demonstrated that changes in Ca^2+^ produced by different glucose and PEP concentrations lead to activation and nuclear translocation of NFAT. For these reasons, we analyzed PEPCK-M contribution to NFAT activation by measuring NFAT activity and NFAT cellular localization in the presence or absence of a potent PEPCK-M inhibitor previously validated in our laboratory (iPEPCK-2; [22]; Figure 4). A reduction in NFAT activity was observed when PEPCK-M was inhibited in the presence of glucose in SW480 cells (Figure 4A), consistent with reduced NFAT activity found in SW480 and HCT116 cell lines upon glucose depletion. In addition, NFAT activity was always lower after PEPCK-M inhibition, indicating that PEPCK-M might have a major role in maintaining NFAT activity (Figure 4A). As reasoned, NFAT targets PTGS2 and IL6 were similarly down-regulated by conditions that reduced PEP concentrations and concomitant cytosolic calcium signaling, namely IPEPCK-2 treatment or glucose deprivation, with an additive down-regulation found in the case of PTGS2 (Figure 4B). Logically, we found that NFAT nuclear localization was mainly observed in the presence of glucose and DMSO and was decreased when cells were glucose starved, or PEPCK-M was inhibited (Figure 4C).

Our results suggest that NFAT activation is modulated in a glucose- and PEPCK-M-dependent manner. For that reason, we aimed to analyze the protein stabilization of c-Myc and c-Myc target genes in conditions of limited PEPCK-M activity. C-Myc phosphorylation at Ser62 was reduced by IPEPCK-2 or by glucose starvation (Figure 4D; quantified in Figure 4E). Although total c-Myc was reduced in the presence of IPEPCK-2, this effect was not statistically significant. Finally, c-Myc targets, such as mGLS, and cSHMT were negatively regulated by IPEPCK-2 consistent with decreased phosphorylation and nuclear localization of c-Myc in these conditions (Figure 4D; quantified in Figure 4C,E). 

## 4. Discussion

The Warburg effect, one of the hallmarks of cancer, allows cancer cells to switch their metabolism to aerobic glycolysis with the objective to increase energy production rates while maintaining anabolism [23,24]. Thus, tumor cells fiercely compete for glucose carbons in the tumor microenvironment with the host tissue and infiltrating immune cells. As a consequence, temporary or locally reduced availability of glucose endures responses in these cells which might contribute to the pathological state of the tumor microenvironment. In a paradigmatic case, T-cells exposed to limiting glucose concentrations cannot activate proper anti-tumoral response [24]. The mechanism for this regulated process was found to implicate the glycolytic intermediate PEP, an inhibitor of ER Ca^2+^-ATP pump SERCA [14]. Therefore, we hypothesized that a similar mechanism would take place in the tumor cell to relay the concentration of glucose and other metabolites in the environment with PEP levels and calcium cell signaling, with consequential gene expression changes that are relevant to tumor biology.

Indeed, our data from two colon carcinoma cell lines confirmed that changes in extracellular glucose concentration exhibited concurrent changes in the levels of PEP and cytosolic calcium. Importantly, changes in intracellular PEP because of direct loading of PEP into the cell or by altering the cataplerotic provision of PEP by the activity of the PEPCK-M pathway using a specific inhibitor, iPEPCK-2, correlated well with changes in calcium levels measured by the surrogate Fluo-4 indicator. Canonical calcium signaling includes calmodulin (CaM) and calcineurin (CaN) pathways, NFAT activation being the most prominent downstream consequence of CaN dependent calcium signaling. Consistently, the NFAT-dependent transcriptional activity of a chimeric luciferase reporter gene was modulated by glucose availability and PEP loading in colon cancer cells, correlating with a predominant localization of NFAT in the nucleus. Also, the inhibition of PEPCK-M exhibited a reduction of NFAT activity and an increase of cytosolic localization of this transcription factor, in accordance with the observed changes in the cytosolic calcium levels. All in all, this suggests that the same mechanisms that impede T-cells from activating anti-tumoral responses in conditions of limited glucose can operate in tumors as the NFAT signaling pathway is reported as a key factor to improve their capacity to effectively compete for these nutrients [16], and in tumor progression and metastasis [25]. Consistently, target genes of NFAT were regulated upon changes in PEP concentrations; cyclooxygenase 2 (PTGS2), an immune-modulator and a negative prognostic factor in colon cancer [26] by promoting tumor progression and metastasis [27]; and IL-6, a cytokine implicated in cancer progression [28], and tumorigenicity [29]. These results suggest that glucose-promoted PEP and/or PEPCK-M activity could have an impact on inflammation, colon cancer progression, and metastasis through the transactivation of PTGS2 and IL6 by the PEP/Ca^2+^/NFAT signaling pathway.

Part of the Ca^2+^ signaling cascade, calmodulin kinase type II gamma (CaMKIIγ), plays an important role in tumor progression of prostate cancer by activation of AKT in a PI3K-independent manner [30], or in the development of colitis-associated cancer through activation of STAT3 [31]. Importantly, CaMKIIγ binds to c-Myc in a calcium-dependent manner [32] and phosphorylates c-Myc at Ser62 thereby increasing its stability and its half-life [11]. Consistently, c-Myc phosphorylation at Ser62 by ionomycin was inhibited only by KN-93, an inhibitor of CaMKIIγ, and not by cyclosporin A. PEP loading increased c-Myc phosphorylation at Ser62 by CaMKIIγ. These results agree with data reported by Ying Gu and collaborators [11] and suggest that c-Myc protein is regulated by PEP/Ca^2+^ by posttranslational mechanisms, independently of calcineurin/NFAT pathway activation. Other studies indicate that c-Myc can be regulated by NFAT transcriptionally [33]. However, we did not observe regulation of c-Myc transcription after changes in PEP levels (data not shown), suggesting cell-to-cell variability in the transcriptional response to NFAT on c-Myc transcription [34]. Interestingly, our data show that total c-Myc protein levels were lower when glucose was lacking, pointing to c-Myc stability regulation by calcium. Concurrently with c-Myc phosphorylation rates, several c-Myc targets relevant to metabolism were also regulated by glucose, and this regulation was PEP-dependent. Moreover, nuclear distribution was also observed in the presence of glucose, consistent with the translocation of phosphorylated c-Myc in these situations. 

Several c-Myc targets modulated by PEP/Ca^2+^ (i.e., Glut1, LDH-A) are associated with clinical colorectal cancer progression [35] and are also induced by K-Ras, commonly mutated in these cancers. Regulation of other targets such as glutaminase (GLS1) and the cytosolic isoform of serine hydroxymethyltransferase (cSHMT) suggest changes in the metabolic layout in agreement with the Warburg effect phenotype [23,36]. This is consistent with the need for double inhibition of c-Myc and PI3K signaling pathways to reduce glucose uptake and glycolytic flux in lymphoma cell lines [37]. In this setting, glucose uptake could regulate c-Myc activation through PEP and calcium, providing a feedback loop into its own metabolism. Consistently, the inhibition of PEPCK-M reduced the amount of PEP and phosphorylated c-Myc in the presence or absence of glucose, accounting for a regulatory axis that is independent of glucose but can promote glucose metabolism.

In this context, it is plausible to view PEP as a rheostat of either glucose carbon flux or cataplerotic fluxes (i.e., PEPCK-M) from other carbons sources such as glutamine, to “inform” on the availability of biosynthetic precursors of the glycolytic pool, such as serine and glycine, to cope with sustenance and growth at various stages of tumor development. Thus, PEPCK-M could regulate calcium signaling, especially in glucose starved conditions where glucose does not contribute to the PEP pool. PCK2 transcription is controlled by the ER stress pathway, downstream of GNC2-PERK-ATF4 [15]. ER is quasi constitutive in tumors, where glucose is low. Therefore, ER-stress could potentiate the PEP/Ca^2+^ axis in a PEPCK-M dependent manner, especially in conditions of limited nutrients for the cell. In fact, PEPCK-M is able to impact on PEP and calcium levels even in the presence of glucose, especially in HCT116 cells. A metabolomics study on HeLa cells (data not shown) showed that PEPCK-M activity contributed glutamine labeled carbons into PEP only under glucose starvation, suggesting that PEPCK-M might influence PEP levels indirectly, possibly by altering the glycolytic flux in the cell. However, the glycolytic flux measured in HCT116 and SW480 cells with [5-^3^H]-glucose clearly indicated that glycolysis was unaffected by PEPCK-M inhibition (Supplementary Figure S1). In addition, glucose consumption or lactate production was not altered by PEPCK-M inhibition (Supplementary Figure S2). Altogether, these results indicate that PEPCK-M does not alter PEP levels indirectly through the modulation of glycolysis, and hint for a contribution of PEPCK-M to the maintenance of the PEP pool in the presence of glucose by fluxing glutamine or other anaplerotic carbons from the TCA cycle, at least in certain types of cancer.

**Figure 5:**
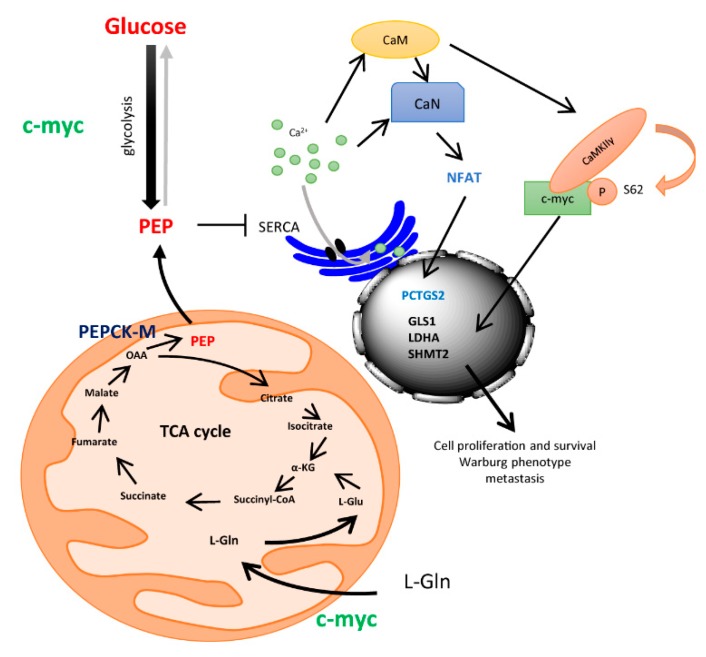
Drawing depicting a model for the integration of ER-stress mediated up-regulation of PEPCK-M and the control of integrative signals downstream of the PEP/Ca^2+^ axis.

To conclude, we propose a model where glucose and PEPCK-M regulate calcium signaling through PEP (Figure 5). Glucose metabolism or cataplerosis from carbons such as glutamine, contribute to maintaining PEP levels, leading to an inhibition of the SERCA pump similarly to T-cells [14]. Cytosolic Ca^2+^ then activates calcineurin (CaN) and calmodulin (CaM) pathways, and in turn, NFAT is translocated into the nucleus, whereas c-Myc is stabilized upon CaMKIIγ phosphorylation at Ser62. NFAT and c-Myc produce activation of expression of several genes implicated in inflammation and metastasis (PTGS2), glucose uptake and glycolysis (Glut1 and HKII), glutamine catabolism (GLS1) and serine and glycine synthesis, and transference of carbons to one-carbon metabolism (cSHMT) that allow for NADPH recycling. This scenario indicates that glucose and PEPCK-M are supporting, through the PEP/Ca^2+^ axis, a proliferative state in tumors, and bring about the importance of this target for cancer metabolism, as exemplified by the efficacy of potent inhibitors of this pathway in pre-clinical studies [22]. 

## Supplementary Materials

The following are available online at https://www.mdpi.com/2073-4409/9/1/18/s1, Figure S1: Glycolytic flux was independent of PEPCK-M activity in human colon carcinoma cells, Figure S2: PEPCK-M activity did not alter glucose consumption and lactate production, Figure S3: Quantitation of WB showed at Figure 3A.

## References

[1] Wiel C., Lallet-Daher H., Gitenay D., Gras B., Le Calvé B., Augert A., Ferrand M., Prevarskaya N., Simonnet H., Vindrieux D. (2014). Endoplasmic reticulum calcium release through ITPR2 channels leads to mitochondrial calcium accumulation and senescence. Nat. Commun..

[2] Rimessi A., Bonora M., Marchi S., Patergnani S., Marobbio C.M., Lasorsa F.M., Pinton P. (2013). Perturbed mitochondrial Ca^2+^ signals as causes or consequences of mitophagy induction. Autophagy.

[3] Xu M., Seas A., Kiyani M., Ji K.S.Y., Bell H.N. (2018). A temporal examination of calcium signaling in cancer- from tumorigenesis, to immune evasion, and metastasis. Cell Biosci..

[4] Monteith G.R., Prevarskaya N., Roberts-Thomson S.J. (2017). The calcium–cancer signalling nexus. Nat. Rev. Cancer.

[5] Shaw J.P., Utz P.J., Durand D.B., Toole J.J., Emmel E.A., Crabtree G.R. (1988). Identification of a putative regulator of early T cell activation genes. Science.

[6] Pan M.-G., Xiong Y., Chen F. (2013). NFAT gene family in inflammation and cancer. Curr. Mol. Med..

[7] Dang C. (1999). V c-Myc target genes involved in cell growth, apoptosis, and metabolism. Mol. Cell. Biol..

[8] Goetzman E.S., Prochownik E.V. (2018). The Role for Myc in Coordinating Glycolysis, Oxidative Phosphorylation, Glutaminolysis, and Fatty Acid Metabolism in Normal and Neoplastic Tissues. Front. Endocrinol. (Lausanne).

[9] Wang R., Dillon C.P., Shi L.Z., Milasta S., Carter R., Finkelstein D., McCormick L.L., Fitzgerald P., Chi H., Munger J. (2011). The transcription factor Myc controls metabolic reprogramming upon T lymphocyte activation. Immunity.

[10] Dang C. (2013). V MYC, metabolism, cell growth, and tumorigenesis. Cold Spring Harb. Perspect. Med..

[11] Gu Y., Zhang J., Ma X., Kim B., Wang H., Li J., Pan Y., Xu Y., Ding L., Yang L. (2017). Stabilization of the c-Myc Protein by CAMKIIγ Promotes T Cell Lymphoma. Cancer Cell.

[12] Bittremieux M., Parys J.B., Pinton P., Bultynck G. (2016). ER functions of oncogenes and tumor suppressors: Modulators of intracellular Ca^2+^ signaling. Biochim. Biophys. Acta Mol. Cell Res..

[13] Cole J.T., Kean W.S., Pollard H.B., Verma A., Watson W.D. (2012). Glucose-6-phosphate reduces calcium accumulation in rat brain endoplasmic reticulum. Front. Mol. Neurosci..

[14] Ho P.-C., Bihuniak J.D., Macintyre A.N., Staron M., Liu X., Amezquita R., Tsui Y.-C., Cui G., Micevic G., Perales J.C. (2015). Phosphoenolpyruvate Is a Metabolic Checkpoint of Anti-tumor T Cell Responses. Cell.

[15] Méndez-Lucas A., Hyroššová P., Novellasdemunt L., Viñals F., Perales J.C. (2014). Mitochondrial phosphoenolpyruvate carboxykinase (PEPCK-M) is a pro-survival, endoplasmic reticulum (ER) stress response gene involved in tumor cell adaptation to nutrient availability. J. Biol. Chem..

[16] Chang C.-H., Qiu J., O’Sullivan D., Buck M.D., Noguchi T., Curtis J.D., Chen Q., Gindin M., Gubin M.M., van der Windt G.J.W. (2015). Metabolic Competition in the Tumor Microenvironment Is a Driver of Cancer Progression. Cell.

[17] Vincent E.E., Sergushichev A., Griss T., Gingras M.C., Samborska B., Ntimbane T., Coelho P.P., Blagih J., Raissi T.C., Choinière L. (2015). Mitochondrial Phosphoenolpyruvate Carboxykinase Regulates Metabolic Adaptation and Enables Glucose-Independent Tumor Growth. Mol. Cell.

[18] Alvarez Z., Hyrossova P., Perales J.C., Alcántara S. (2016). Neuronal Progenitor Maintenance Requires Lactate Metabolism and PEPCK-M-Directed Cataplerosis. Cereb. Cortex.

[19] Balsa-Martinez E., Puigserver P. (2015). Cancer Cells Hijack Gluconeogenic Enzymes to Fuel Cell Growth. Mol. Cell.

[20] Méndez-Lucas A., Duarte J.A.G., Sunny N.E., Satapati S., He T., Fu X., Bermúdez J., Burgess S.C., Perales J.C. (2013). PEPCK-M expression in mouse liver potentiates, not replaces, PEPCK-C mediated gluconeogenesis. J. Hepatol..

[21] Leithner K., Triebl A., Trötzmüller M., Hinteregger B., Leko P., Wieser B.I., Grasmann G., Bertsch A.L., Züllig T., Stacher E. (2018). The glycerol backbone of phospholipids derives from noncarbohydrate precursors in starved lung cancer cells. Proc. Natl. Acad. Sci. USA.

[22] Aragó M., Moreno-Felici J., Abás S., Rodríguez-Arévalo S., Hyroššová P., Figueras A., Viñals F., Pérez B., Loza M.I., Brea J. (2020). Pharmacology and preclinical validation of a novel anticancer compound targeting PEPCK-M. Biomed. Pharmacother..

[23] Vander Heiden M.G., Cantley L.C., Thompson C.B. (2009). Understanding the Warburg effect: The metabolic requirements of cell proliferation. Science.

[24] Pearce E.L., Poffenberger M.C., Chang C.-H., Jones R.G. (2013). Fueling immunity: Insights into metabolism and lymphocyte function. Science.

[25] Tie X., Han S., Meng L., Wang Y., Wu A. (2013). NFAT1 Is Highly Expressed in, and Regulates the Invasion of, Glioblastoma Multiforme Cells. PLoS ONE.

[26] Cetin M., Buyukberber S., Demir M., Sari I., Sari I., Deniz K., Eser B., Altuntas F., Camcı C., Öztürk A. (2005). Overexpression of cyclooxygenase-2 in multiple myeloma: Association with reduced survival. Am. J. Hematol..

[27] Al-Salihi M.A., Terrece Pearman A., Doan T., Reichert E.C., Rosenberg D.W., Prescott S.M., Stafforini D.M., Topham M.K. (2009). Transgenic expression of cyclooxygenase-2 in mouse intestine epithelium is insufficient to initiate tumorigenesis but promotes tumor progression. Cancer Lett..

[28] Nagasaki T., Hara M., Nakanishi H., Takahashi H., Sato M., Takeyama H. (2014). Interleukin-6 released by colon cancer-associated fibroblasts is critical for tumour angiogenesis: Anti-interleukin-6 receptor antibody suppressed angiogenesis and inhibited tumour–stroma interaction. Br. J. Cancer.

[29] Lin J.-T., Wang J.-Y., Chen M.-K., Chen H.-C., Chang T.-H., Su B.-W., Chang P.-J. (2013). Colon cancer mesenchymal stem cells modulate the tumorigenicity of colon cancer through interleukin 6. Exp. Cell Res..

[30] Rokhlin O., Taghiyev A.F., Bayer K.U., Bumcrot D., Kotelianski V.E., Glover R.A., Cohen M.B. (2007). Calcium/calmodulin-dependent kinase II plays an important role in prostate cancer cell survival. Cancer Biol. Ther..

[31] Ma X., Meng Z., Jin L., Xiao Z., Wang X., Tsark W.M., Ding L., Gu Y., Zhang J., Kim B. (2017). CAMK2γ in intestinal epithelial cells modulates colitis-associated colorectal carcinogenesis via enhancing STAT3 activation. Oncogene.

[32] Raffeiner P., Schraffl A., Schwarz T., Röck R., Ledolter K., Hartl M., Konrat R., Stefan E., Bister K. (2017). Calcium-dependent binding of Myc to calmodulin. Oncotarget.

[33] Singh G., Singh S.K., König A., Reutlinger K., Nye M.D., Adhikary T., Eilers M., Gress T.M., Fernandez-Zapico M.E., Ellenrieder V. (2010). Sequential Activation of NFAT and c-Myc Transcription Factors Mediates the TGF-β Switch from a Suppressor to a Promoter of Cancer Cell Proliferation. J. Biol. Chem..

[34] Mognol G.P., de Araujo-Souza P.S., Robbs B.K., Teixeira L.K., Viola J.P.B. (2012). Transcriptional regulation of the *c-Myc* promoter by NFAT1 involves negative and positive NFAT-responsive elements. Cell Cycle.

[35] Graziano F., Ruzzo A., Giacomini E., Ricciardi T., Aprile G., Loupakis F., Lorenzini P., Ongaro E., Zoratto F., Catalano V. (2017). Glycolysis gene expression analysis and selective metabolic advantage in the clinical progression of colorectal cancer. Pharm. J..

[36] Burns J.S., Manda G. (2017). Metabolic Pathways of the Warburg Effect in Health and Disease: Perspectives of Choice, Chain or Chance. Int. J. Mol. Sci..

[37] Broecker-Preuss M., Becher-Boveleth N., Bockisch A., Dührsen U., Müller S. (2017). Regulation of glucose uptake in lymphoma cell lines by c-MYC- and PI3K-dependent signaling pathways and impact of glycolytic pathways on cell viability. J. Transl. Med..

